# Global trends and future projections of cervical cancer burden: an integrated analysis of GBD 2021, UN population and WHO HPV vaccination data

**DOI:** 10.3389/fpubh.2026.1702186

**Published:** 2026-01-26

**Authors:** Dongxuan Shao, Ping Wu, Huici Jiang, Zhijie Wang

**Affiliations:** 1 Department of Gynecology and Obstetrics, Shanghai Eighth People's Hospital, Shanghai, China; 2 Department of Gynecology and Obstetrics, Shanghai Fourth People's Hospital, School of Medicine, Tongji University, Shanghai, China

**Keywords:** cervical cancer, global burden of disease, HPV vaccination, age-standardized incidence, age-standardized mortality rates

## Abstract

**Background:**

Cervical cancer remains a leading cause of morbidity and mortality among women, disproportionately affecting low- and middle-income countries (LMICs). We sought to: (1) characterize temporal and geographic patterns of cervical cancer burden (1990–2021), with a focus on age-related differences; (2) identify attributable risk factors for cervical cancer, emphasizing the impact of HPV vaccination; (3) forecast cervical cancer burden through 2050.

**Methods:**

We combined: (a) age-specific female population estimates (UN World Population Prospects 2024), (b) cervical cancer incidence, mortality and disability-adjusted life-years (DALYs) from Global Burden of Disease (GBD) 2021, and (c) HPV vaccination coverage (WHO). We calculated age-standardized incidence (ASIR), mortality (ASMR), and DALYs rate (ASDR) using the WHO world standard population. Stratified analyses were performed by Socio-demographic Index (SDI) category and 5-year age groups. Future burdens were projected under current intervention coverage.

**Findings:**

From 1990 to 2021, global ASIR, ASMR and ASDR declined by 15, 31 and 32%, respectively, yet absolute cases rose due to population growth and ageing. The greatest burdens remain in low-SDI regions, especially Southern Sub-Saharan Africa, which uniquely saw rising ASIR and ASMR. Women aged 55–59 bear the highest rates, while young women (15–39) experienced a small but significant incidence increase in 92 countries (notably Russia, Brazil and China). Unsafe sex and smoking accounted for the majority of cervical cancer DALYs. A profound disparity in HPV vaccine coverage persists between high- and low-SDI regions. Projections to 2050 indicate a continued rise in absolute case numbers, despite modest declines in age-standardized rates (ASRs).

**Conclusion:**

While ASRs show improvement, the growing absolute burden and profound geographic inequities highlight an urgent public health challenge. Accelerating the scale-up of HPV vaccination, screening, and other preventive measures, with a strategic focus on LMICs, is critical to achieving the WHO elimination targets for cervical cancer.

## Introduction

Cervical cancer is a major global public health challenge, ranking as the fourth most common malignancy among women, with significant morbidity and mortality, especially in low- and middle-income countries (LMICs) (1–3). Effective HPV vaccination and organized screening programs can significantly prevent cervical cancer, yet women in resource-limited settings remain disproportionately affected due to inadequate access to these services (4). This reflects differences in socioeconomic conditions and healthcare policies (5). Notably, age-standardized incidence (ASIR) and mortality rate (ASMR) are highest in Sub-Saharan Africa and parts of Asia and Latin America. While high-income countries have seen declines in incidence due to widespread screening and vaccination, many LMICs—particularly among women aged 15-49—are experiencing stable or rising trends (6).

In response to these challenges, the WHO’s initiative to eliminate cervical cancer aims to reduce incidence rates to fewer than 4 cases per 100,000 women per year by 2030. However, realizing this target requires reliable epidemiological data to monitor progress and identify high-risk populations. Research indicates that, without increased coverage of preventive measures, the cervical cancer burden in many LMICs will remain high due to factors like population growth (7). Additionally, over the past two decades, demographic structure changes (such as population growth, population aging) and HPV vaccination have exerted a significant impact on the global burden of cervical cancer. A comprehensive understanding of the current situation under demographic structure changes and HPV vaccination, is essential for informing public health policies and optimizing resource allocation. However, previous studies have largely relied on single data sources, such as the Global Burden of Disease (GBD) database, lacking integration of multi-source data and systematic projections of future burdens (3, 5).

Therefore, this study innovatively integrates three authoritative data sources (GBD 2021, UN Population, and WHO HPV vaccination coverage). GBD supply corresponding data of incidence, mortality, and Disability-Adjusted Life Years (DALYs), UN Population provide the baseline population of women across age groups, while HPV vaccination data serve as the key intervention variable influencing future disease trend projections. Through this integrated analytical framework, this study aims to: (1) characterize temporal and geographic patterns of cervical cancer burden (1990–2021), with a focus on age-related differences; (2) identify attributable risk factors for cervical cancer, emphasizing the impact of HPV vaccination; (3) forecast cervical cancer burden through 2050.

## Materials and methods

### Data sources

Data were obtained from three primary sources:

1. Age-specific global female population estimates (1990–2021) from the United Nations Population Division’s World Population Prospects 2024 (https://population.un.org/wpp/Download/).
2. Cervical cancer incidence, mortality, DALYs, and risk factor data (1990–2021) for 204 countries/territories from the GBD 2021 database (https://vizhub.healthdata.org/gbd-results/), with stratification by location and age.
3. HPV vaccination Coverage (Immunization data) from WHO (https://immunizationdata.who.int/).

We obtained data on the number of women in specific age groups worldwide from the Population Division World Population Prospects 2024 for the years 1990–2021. The HPV vaccination coverage data for different regions were sourced from Immunization data from the WHO. Finally, we extracted data on the number and rate of incidence and deaths from cervical cancer, along with the risk factors for DALYs, for 204 countries and regions from GBD 2021, and conducted stratified analysis by location and age.

### Statistical analysis

Age-standardized rates (ASRs): adjusted using the WHO World Standard Population via the following formulas:


$$\mathrm{ASR}=\frac{\sum_{i}(a_{i}\times w_{i})}{\sum_{i}w_{i}}\times 100000$$

where $a_{i}$ is the age specific rate in age group 𝑖, and $w_{i}$ is the weight of the WHO World Standard Population in age group 𝑖.

DALYs: Calculated as the sum of years of life lost (YLLs) and years lived with disabilities (YLDs), where YLLs represent the years lost due to premature death, and YLDs are assessed based on the health status of survivors (8).

We calculated ASRs using the WHO World Standard Population. Additionally, we used linear regression-based Estimated Annual Percentage Change (EAPC) calculations to quantify the overall trend from 1990 to 2021.

It is worth noting that in the age stratification, we adopted a more detailed breakdown with 5-year intervals. Additionally, we utilized the Socio-demographic Index (SDI) provided by GBD 2021 for regional analysis, which is derived from indicators such as per capita income, education levels, and fertility rates, categorizing the 204 countries and regions into five groups: high, high-middle, middle, low-middle, and low, based on SDI. Furthermore, the DALYs used to examine risk factors represent a composite measure of disease burden.

To enhance the robustness of the prediction results and capture different drivers of disease burden, this study simultaneously employed two predictive models: the Bayesian Age–Period–Cohort (BAPC) model to analyze age/cohort effects, and the Autoregressive Integrated Moving Average (ARIMA) model to capture temporal autocorrelation, and utilize backward prediction to calculate metrics such as the root mean square error (RMSE) to evaluate the model’s predictive performance. This study performed approximate Bayesian inference using the BAPC package in R and its dependent Integrated Nested Laplace Approximation (INLA) framework to predict ASIR, ASMR, and ASDR, and their probability intervals for cervical cancer from 2022 to 2050. Additionally, the auto.arima function in the forecast package of R was employed to automatically select the optimal ARIMA model based on the criterion of minimizing the Akaike Information Criterion (AIC), thereby obtaining the predicted data of new cases and deaths of cervical cancer from 2022 to 2050, the proportion of new cases and deaths of cervical cancer among people aged 40 years and above from 2022 to 2050, and the predicted data of DALYs. It should be noted that the projected data obtained from the ARIMA model are point estimates. Additionally, we obtained estimates from the International Agency for Research on Cancer (IARC) for the number of new cervical cancer cases and deaths between 2022 and 2050.^1^

To obtain the ratio of cervical cancer aged ≥ 40 years, we obtained the number of cervical cancer cases across all ages and aged ≥ 40 years worldwide from 1990 to 2021 using GBD data. We then calculated the proportion of cervical cancer cases aged≥ 40 years among all-age cervical cancer cases, which is referred to as “Cervical cancer patients.” Second, using the same method, we calculated the proportion of female cancer cases aged > 40 years among female cancer cases across all ages, which is referred to as “All female patients with cancer.” Finally, we obtained the total number of females worldwide and the number of females aged ≥40 years from 1990 to 2021 via UN data. We further calculated the proportion of females aged ≥ 40 years among the total global female population, which is referred to as “World female population”. Statistical analyses were conducted in R v4.2.1 (R Foundation, Vienna).

## Results

### Incidence, mortality, and DALYs of cervical Cancer by regions

**Figure 1:**
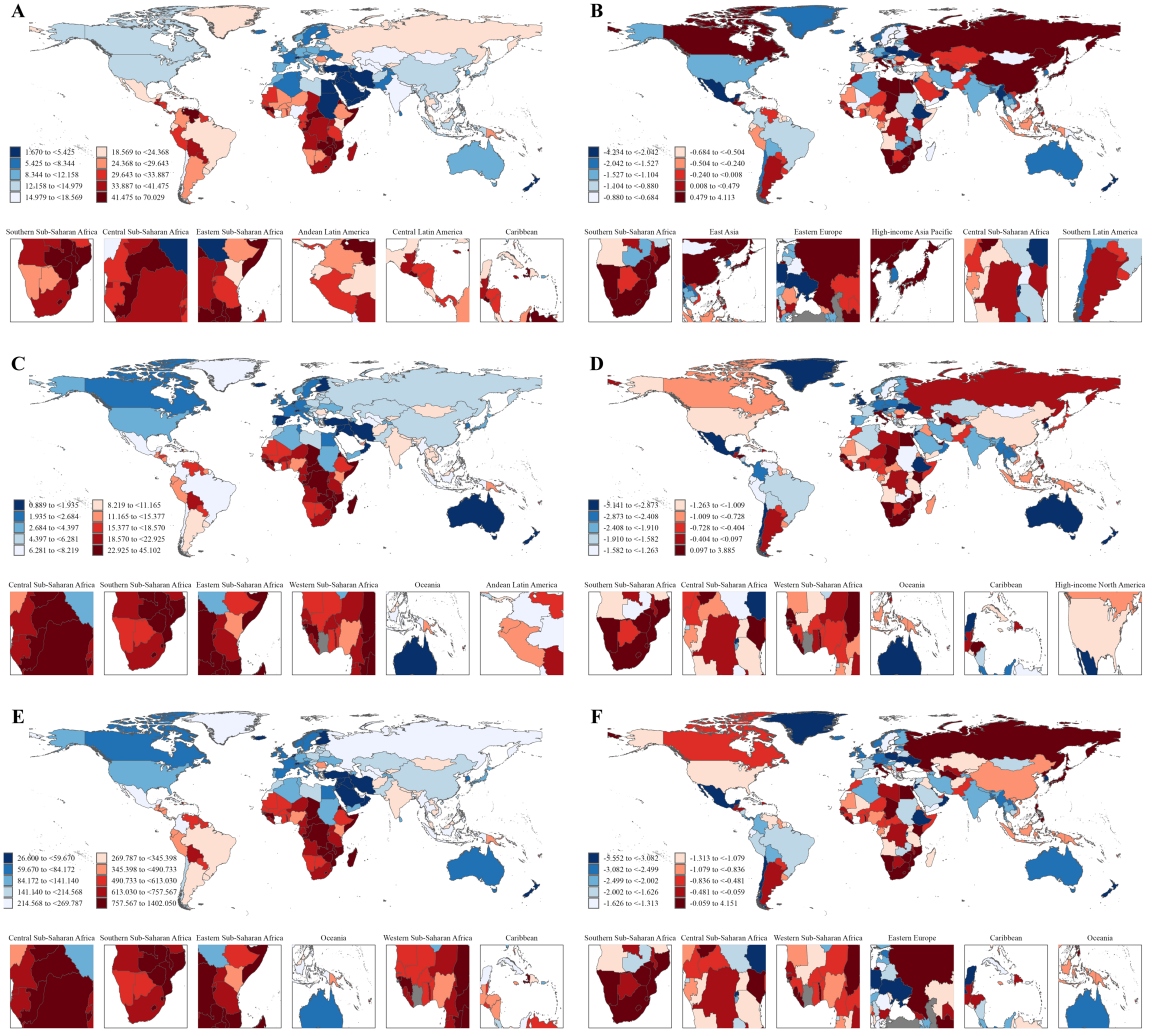
Global and regional burden of cervical cancer by incidence **(A)**, deaths **(C)**, and DALYs **(E)** in 2021, as well as EAPC of incidence **(B)**, deaths **(D)**, and DALYs **(F)** from 1990–2021.

According to the GBD 2021 database (Table 1), there are approximately 667,426 new cases of cervical cancer diagnosed globally each year. In terms of new cases across GBD regions, Southern Sub-Saharan Africa, Central Sub-Saharan Africa, Eastern Sub-Saharan Africa, Andean Latin America, Central Latin America, Caribbean, Oceania, Western Sub-Saharan Africa, Southern Latin America, and Tropical Latin America ranked top 10 regions in ASIR. These regions also comprised the top 10 for ASMR and ASDR, despite Tropical Latin America being replaced by South Asia (Table 1; Figures 1A,C,E).

**Table 1:** Age-standard incidence (ASIR), mortality (ASMR), DALYs, and estimated percentage change (EAPC) of cervical cancer from 1990 to 2021, by GBD regions and SDI level.

GBD regions and SDI level	ASIR (per 100,000)			ASMR (per 100,000)			DALYs (per 100,000)		
	1990	2021	EAPC	1990	2021	EAPC	1990	2021	EAPC
Global	18.11	15.32	−0.54	9.68	6.62	−1.27	330.11	226.28	−1.27
GBD regions									
Central Sub-Saharan Africa	39.39	38.00	−0.17	28.67	25.10	−0.47	950.65	813.59	−0.54
Eastern Sub-Saharan Africa	45.69	33.45	−1.34	32.97	21.68	−1.67	1117.94	709.49	−1.81
Southern Sub-Saharan Africa	29.89	42.40	**1.89**	17.43	23.90	**1.71**	600.46	788.82	**1.71**
Western Sub-Saharan Africa	26.20	24.11	−0.24	18.33	15.57	−0.48	608.91	490.75	−0.68
North Africa and Middle East	5.98	4.72	−0.73	3.75	2.55	−1.39	131.01	80.04	−1.59
Andean Latin America	33.19	29.79	−0.65	20.85	14.02	−1.57	667.44	431.74	−1.71
Caribbean	31.51	27.58	−0.45	16.36	12.31	−0.88	561.02	433.17	−0.80
Central Latin America	41.85	28.89	−1.58	20.38	9.52	−2.78	627.39	315.97	−2.55
Tropical Latin America	20.08	20.27	−0.85	13.25	8.38	−1.78	422.16	285.57	−1.64
Central Asia	18.21	14.17	−0.45	9.53	6.33	−1.06	318.71	213.84	−1.05
Central Europe	21.66	15.93	−1.10	10.15	6.02	−1.85	344.13	191.83	−2.05
Eastern Europe	14.94	16.49	**0.31**	7.58	5.50	−1.22	239.05	200.77	−0.70
East Asia	12.16	13.40	**0.73**	7.10	4.68	−1.12	231.92	151.15	−1.13
Oceania	34.17	27.31	−0.80	19.77	15.27	−0.82	654.65	499.47	−0.86
Southeast Asia	18.06	15.17	−0.78	10.70	7.45	−1.32	362.63	241.92	−1.48
South Asia	23.70	15.49	−1.48	15.97	8.72	−2.06	552.66	285.98	−2.23
Australasia	17.18	8.51	−1.92	5.34	1.85	−3.21	178.43	61.68	−3.13
High-income Asia Pacific	11.85	11.03	**0.02**	4.27	2.53	−1.67	133.71	87.68	−1.26
High-income North America	19.65	12.69	−1.34	3.96	2.64	−1.00	133.17	92.68	−1.05
Southern Latin America	24.43	22.80	−0.22	12.10	8.56	−1.10	417.99	296.76	−1.08
Western Europe	14.25	8.71	−1.27	4.70	2.29	−2.02	148.36	72.71	−2.02
SDI level									
High SDI	13.37	10.30	−1.41	5.03	2.62	−2.05	163.46	86.41	−1.99
High-middle SDI	16.43	13.27	−0.21	6.83	4.59	−1.20	222.34	152.90	−1.12
Middle SDI	18.09	15.94	−0.47	10.47	6.72	−1.50	342.74	218.95	−1.52
Low-middle SDI	22.48	17.79	−0.78	14.70	9.71	−1.35	505.86	321.36	−1.48
Low SDI	34.34	25.47	−1.17	24.51	16.36	−1.49	833.33	535.11	−1.64

Bolded values indicate a positive EAPC.

Regarding SDI level, whether in 1990 or 2021, ASIR, ASMR, and ASDR were negatively correlated with SDI (Supplementary Figures S1A-C; Supplementary Table S1). Concurrently, as the SDI increases, the proportion of patients aged 40 and above rises, with this trend being most pronounced in deaths and DALYs (Supplementary Figures S1D–F; Supplementary Table S1).

From the GBD 2021 data, historical trends of cervical cancer are evident (Table 1, Figures 1B,D,F). Between 1990 and 2021, the ASIR, ASMR, and ASDR for cervical cancer globally all showed a downward trend. Almost all GBD regions and various SDI-related areas have observed a decrease in ASIR, ASMR, and ASDR (Figures 1B,D,F; Supplementary Figures S1A–C). Furthermore, the majority of GBD regions, except for Southern Sub-Saharan Africa, Eastern Europe, and East Asia, have experienced a decline in ASIR. Notably, Southern Sub-Saharan Africa has shown an increase in ASIR, ASMR, and ASDR. Meanwhile, the reduction in ASIR for High-middle SDI and Middle SDI regions is significantly lower than that of other areas (percentage change, −0.01% and −0.12%, respectively).

### Disease burden of cervical cancer by age group

**Figure 2:**
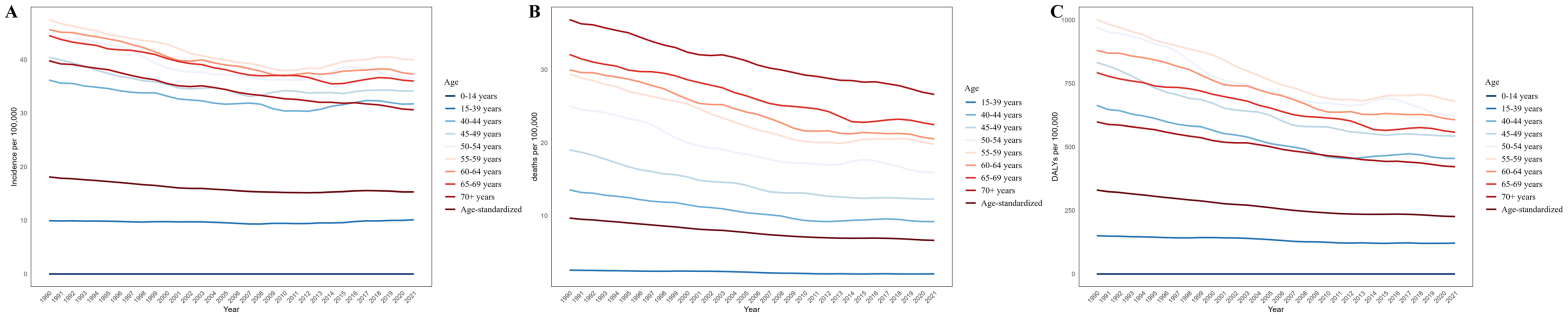
Trends of incidence **(A)**, deaths **(B)**, and DALYs **(C)** from 1990 to 2021 by age group.

Cervical cancer predominantly affects individuals aged 40 and above, especially those aged 50–69, with the highest incidence and DALYs in the 55–59 age group, while the death rate increases with age (Table 2; Figure 2). Furthermore, combining GBD 2021 data and UN population data, as shown in Supplementary Figure S2A, the proportion of cervical cancer patients aged 40 and above rose from 73.7% in 1990 to 77.8% in 2021. This proportion is significantly higher than that of all cancers in women aged 40 and above and higher than the global proportion of women aged 40 and above.

**Table 2:** Age-standard incidence (ASIR), mortality (ASMR), DALYs, and total percentage change (TPC) of cervical cancer from 1990 to 2021, by age group.

Age	ASIR (per 100,000)			ASMR (per 100,000)			DALYs (per 100,000)		
	1990	2021	TPC	1990	2021	TPC	1990	2021	TPC
<40	9.95	10.11	**0.02**	2.56	2.04	−0.20	150.63	121.22	−0.20
15–19	0.93	0.92	−0.01	0.27	0.21	−0.23	20.28	15.81	−0.22
20–24	2.59	2.75	**0.06**	0.72	0.62	−0.14	50.13	43.55	−0.13
25–29	7.94	7.58	−0.05	1.78	1.40	−0.22	115.77	91.29	−0.21
30–34	18.03	16.12	−0.11	4.08	2.86	−0.30	244.53	173.48	−0.29
35–39	27.28	24.13	−0.12	7.81	5.33	−0.32	425.46	293.67	−0.31
40–69									
40–44	36.19	31.75	−0.12	13.53	9.19	−0.32	663.66	454.90	−0.31
45–49	40.41	34.15	−0.15	19.00	12.27	−0.35	832.60	543.09	−0.35
50–54	44.64	36.61	−0.18	25.01	15.90	−0.36	971.85	623.13	−0.36
55–59	**47.48**	**39.94**	−0.16	29.40	19.81	−0.33	**1001.61**	**680.46**	−0.32
60–64	45.60	37.31	−0.18	29.97	20.52	−0.32	880.30	606.60	−0.31
65–69	44.49	36.01	−0.19	32.06	22.45	−0.30	793.06	557.84	−0.30
70+	39.79	30.64	−0.23	**36.85**	**26.60**	−0.28	598.58	422.14	−0.29

Bolded values denote either a positive TPC growth or that the corresponding age group is at the peak level.

Additionally, regarding age, we note an annual increase of 0.02% in the incidence among the 15–39 age group, which may be attributed to the annual increase of 0.06% in the 20–24 age group. The 20–24 age group also shows inconsistencies in deaths and DALYs compared to other age groups, with a significantly lower annual reduction (Table 2). Upon further analysis of the burden of cervical cancer across different age groups in various SDI level regions (Table 3), we find that incidence has decreased across all age groups in Low SDI and High SDI regions. However, Low-Middle SDI, Middle SDI, and High-Middle SDI regions show an increase in incidence among the 15–39 age group. Particularly, significant increases in every 5-year age interval within the 20–39 range (a similar pattern is observed in the 40–54 age group) were found in High-Middle SDI regions.

**Table 3:** Incidence, and total percentage change (TPC) of cervical cancer from 1990 to 2021, by age group and SDI level.

Age	Low SDI			Low-middle SDI			Middle SDI			High-middle SDI			High SDI		
	1990	2021	TPC	1990	2021	TPC	1990	2021	TPC	1990	2021	TPC	1990	2021	TPC
<40	12.96	11.03	−0.15	9.39	9.45	**0.01**	8.23	10.04	**0.22**	7.68	10.77	**0.40**	15.71	9.83	−0.37
15–19	1.68	1.52	−0.10	1.18	1.07	−0.09	0.88	0.74	−0.16	0.53	0.47	−0.11	0.59	0.30	−0.50
20–24	4.57	4.47	−0.02	3.01	3.15	**0.05**	2.22	2.20	−0.01	1.61	1.66	**0.04**	3.01	1.54	−0.49
25–29	11.80	10.69	−0.09	7.74	7.70	0.00	6.36	6.85	**0.08**	5.39	5.96	**0.11**	12.73	7.13	−0.44
30–34	22.29	19.65	−0.12	16.76	15.67	−0.07	15.51	15.59	**0.01**	13.36	15.24	**0.14**	27.23	16.08	−0.41
35–39	39.74	30.71	−0.23	28.52	24.26	−0.15	24.65	23.51	−0.05	20.30	23.75	**0.17**	33.85	20.31	−0.40
40–69															
40–44	60.16	43.04	−0.28	44.20	34.24	−0.23	34.96	32.10	−0.08	26.10	29.33	**0.12**	33.47	22.01	−0.34
45–49	77.11	52.12	−0.32	52.60	37.90	−0.28	39.49	35.19	−0.11	27.66	29.92	**0.08**	31.43	21.90	−0.30
50–54	95.92	62.05	−0.35	63.11	43.67	−0.31	46.26	38.07	−0.18	30.24	31.20	**0.03**	29.62	21.20	−0.28
55–59	105.96	74.57	−0.30	66.68	50.98	−0.24	49.99	42.59	−0.15	32.85	32.26	−0.02	32.00	21.71	−0.32
60–64	106.70	76.10	−0.29	64.58	46.96	−0.27	46.97	40.80	−0.13	34.15	30.34	−0.11	32.79	20.41	−0.38
65–69	99.06	73.60	−0.26	56.72	46.11	−0.19	46.15	40.48	−0.12	36.92	29.43	−0.20	35.02	20.30	−0.42
70+	79.56	66.74	−0.16	47.14	41.41	−0.12	45.03	35.81	−0.20	34.89	25.83	−0.26	33.81	19.71	−0.42

Bolded values denote a positive TPC growth.

Further analysis of the increase in incidence among the 15–39 age group reveals that, out of 194 countries and regions globally, 92 countries or territories experienced an increase in incidence in this age group (Supplementary Table S1). It was led by the following countries: Russian Federation, Lesotho, Kingdom of Eswatini, Zimbabwe, Italy, Japan, Bolivarian Republic of Venezuela, Namibia, Brazil, and China, with varying sizes of percentage change. Among these 92 countries in 2021, 4 High SDI countries reported a total of 3,013 new cases in the 15–39 age group; 17 Low SDI countries reported 8,572 new cases; 26 Low-Middle SDI countries reported 11,273 new cases; 23 Middle SDI countries reported 20,175 new cases; and 22 High-Middle SDI countries reported 31,339 new cases in the 15–39 age group.

### Risk factors for cervical cancer DALYs

**Figure 3:**
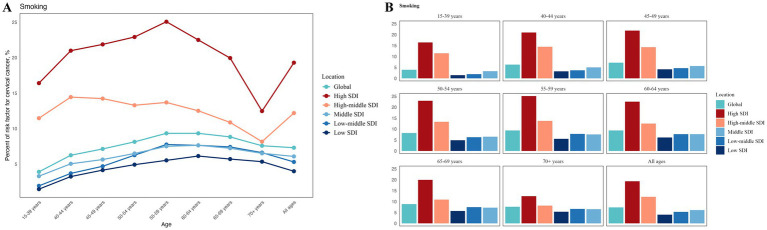
Proportion of smoking-attributable cervical cancer risk across GBD SDI locations and age groups: **(A)** a line plot of its trend across continuous ages, and **(B)** grouped bar plots of its stratified distributions by discrete age ranges.

According to the GBD 2021 data, the primary risk factors for cervical cancer DALYs identified are unsafe sex and smoking. We evaluated the proportion of DALYs due to cervical cancer attributed to smoking across different age groups and SDI. The results, illustrated in Figure 3, indicate that smoking contributes the highest proportion to cervical cancer DALYs among individuals in high SDI and high-middle SDI regions. Furthermore, in most SDI categories, the smoking rate is notably high among the 55–59 age group. It is noteworthy that in high SDI and high-middle SDI regions, the age group with the lowest smoking proportion is those aged 70 and above, whereas in other SDI categories and globally, the lowest smoking proportion is found among the 15–39 age group.

The preventive measure for the risk factor of unsafe sexual behavior is HPV vaccination. We retrieved HPV vaccination coverage data from WHO for various regions from 2010 to 2023. It is evident that high SDI regions, such as the Region of the Americas and the European Region, have achieved HPV vaccination coverage of 30–77%, while medium and low SDI regions, such as the Eastern Mediterranean Region, South-East Asia Region, and Western Pacific Region, report much lower coverage rates of only 0–16%. In 2018, the WHO initiated a global call to action to eliminate cervical cancer, which is reflected in the increase in HPV vaccination coverage across various regions, particularly in Africa, where coverage significantly rose from 1–8% in 2017 to 15–40% (Supplementary Figure S3). Similarly, the COVID-19 pandemic, which began in 2020 globally, has led to a decline in HPV vaccine coverage in many regions worldwide.

### Future burden of cervical cancer

**Figure 4:**
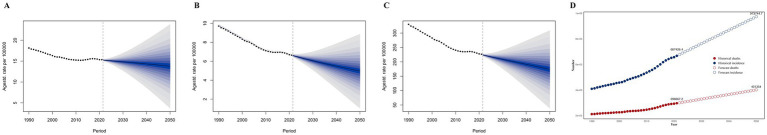
Age-standard incidence rates **(A)**, mortality rates **(C)**, DALYs (C), new cases and deaths **(D)** of cervical cancer globally over time, with projections until 2050.

The ASIR, ASMR, and ASDR are projected to show a potential decline. The ASIR decreased from 15.3 per 100,000 in 2021 to 13.77 per 100,000 in 2050, the ASMR dropped from 6.62 per 100,000 in 2021 to 4.95 per 100,000, and the ASDR fell from 226.28 per 100,000 in 2021 to 173.80 per 100,000 (Figure 4). On the other hand, cervical cancer incidences are projected to rise from 667,000 in 2021 to 973,000 by 2050, deaths are expected to increase from 296,000 to 401,000 (Figure 4D), although the proportion of patients aged 40 and above may not change significantly (Supplementary Figure S4A). IARC projections indicate that by 2050, there will be 948 k new cervical cancer cases and 543 k deaths (Supplementary Figure S5).

## Discussion

In our study, we systematically analyzed global trends in cervical cancer incidence and mortality by integrating data from the GBD 2021 databases, alongside UN population data and WHO HPV vaccination coverage statistics. Our analysis corroborates the dual narrative of cervical cancer control: substantial progress in age-standardized indicators, yet a growing absolute burden propelled by demography and persistent inequities.

Globally, cervical cancer remains one of the major burdens among gynecological cancers. Expansion of cytology-based screening, HPV DNA testing, and (in higher-income settings) prophylactic vaccination has lowered incidence and mortality in women born after the 1960s (9, 10). Improved HIV management in Eastern and Southern Africa may have moderated, though not reversed, the regional epidemic (11). The steepest declines were observed in high-SDI countries and low-SDI countries. Despite those, the global incidence of newly diagnosed cervical cancer cases continues to rise. In 2022, it ranked 8th and 9th in the number of new cases and deaths across all cancers, respectively, and ranked 4th in female cancers (6, 12). Increases in population size and changes in the population aging structure can explain the paradox of a declining ASIR alongside a rising total number of new cases. From 1990 to 2021, the cumulative growth rate of new cases was 62.9%, while the population growth rate was 48.9%. Thus, the risk ratio attributed to population growth was approximately 77.7% [(48.9%/62.9%) × 100%].

We found that the 10.4% increase in the global population aged ≥40 years stems from population aging, contributing 16.5% to the growth in new cases among those aged ≥40 years [(10.4%/62.9%) × 100%]. In this study, ageing may help explain the disproportionate decrease in cervical cancer incidence in middle and high-middle SDI countries (PC −0.12 and −0.01, much higher than those in other SDI level country); the remainder stems from incomplete vaccine coverage and limited screening in low-resource settings (13).

Regional disparities are still evident, with particularly high ASIR and ASMR in Sub-Saharan Africa, Latin America, and Oceania. These significant regional variations are closely related to economic and social development levels, unequal distribution of medical resources, human papillomavirus genotypes, and disparities in health service availability, corroborating existing studies (14, 15). Notably, Southern Sub-Saharan Africa has seen a substantial increase in ASIR, ASMR, and DALYs, necessitating greater international collaboration.

An emerging concern is the rising incidence of cervical cancer among young women, which calls for increased attention and action to address this troubling trend. The modest yet consistent uptick among aged 15–39 years—marked in Russia, Southern Africa, Brazil and China—may reflect earlier sexual debut, sub-optimal vaccine catch-up programs, and changing HPV genotype distribution (16–18). Because invasive disease at <40 years threatens fertility and long-term productivity, tailored prevention (gender-neutral vaccination, self-sampling) is needed (19). Furthermore, among these 92 countries rising incidence of cervical cancer among young women, middle SDI countries constitute the majority (49/92) and also account for the largest share of new cases (62,787/74,373). These countries are characterized by notable population growth but low HPV vaccine coverage. In recent years, HPV vaccination rates in Brazil and South Africa have gradually risen from approximately 10% in 2016 to around 80%. China has launched cervical cancer elimination initiatives, with HPV vaccination coverage gradually reaching 10–20% (7). In contrast, HPV vaccination is not yet included in Russia’s public health policy framework. Therefore, in diverse middle SDI countries and regions, it is crucial to expand HPV coverage and promote public education on standardized sexual behavior, as these efforts can effectively mitigate the younger age trend of cervical cancer.

In our analysis of cervical cancer risk factors, unsafe sexual behavior has long been recognized as a dominant contributor to the disease burden (17, 20). However, this study specifically highlights that smoking is an intervenable yet frequently overlooked risk factor. Particularly in high-SDI and high-middle-SDI regions, smoking contributes significantly to cervical cancer DALYs, accounting for approximately 20 and 10%, respectively. This reflects the potential impact of regional development levels on risk exposure patterns, indicating that tobacco control policies and health education must be integrated into comprehensive cervical cancer prevention programs (21).

The analysis across different SDI regions reveals substantial disparities in economic development and social resource allocation. High-SDI regions exhibit higher HPV vaccination coverage compared to low and middle-SDI regions. This disparity underscores that the success of HPV vaccination promotion is primarily influenced by economic factors, policy support, enhanced community awareness, and the development of primary healthcare systems (22). Since 2018, the WHO has been advocating for the global initiative to “eliminate cervical cancer,” which has shown initial success in improving vaccination coverage in low- and middle-income regions, particularly in Africa, and is expected to significantly impact cervical cancer trends in these areas in the future. However, overall inequality across SDI regions remains severe, highlighting the need for sustained global collaboration and financial investment as essential conditions for achieving equitable vaccine access (23, 24).

Finally, our projections indicate a substantial increase in the incidence, mortality, and DALYs of cervical cancer globally by 2050, with over 900,000 new cases and more than 400,000 deaths. These findings are similar to projections from the IARC. Addressing this pressing situation hinges on expanding vaccination coverage and continuing the promotion of screening programs (25). It is particularly crucial to strengthen resource allocation and healthcare system development in developing countries; otherwise, we will continue to face significant disease burden pressures in the future.

## Limitations

Despite the strengths of this study, limitations exist. Certain issues may introduce bias into the research findings. For instance, GBD data are derived from mathematical model estimates, regions with low social development indices may experience underreporting of data, and the research may be subject to the ecological fallacy. Second, the predictions in this study rely on the fundamental assumptions of the BAPC and ARIMA models, which may become invalid during major policy shifts or sudden public health emergencies. Although we employed both models to enhance robustness and partially validated the predictions using IARC forecast data, their predictive efficacy remains constrained by the quality and completeness of historical data. Additionally, the use of the DALY methodology might oversimplify interactions among various risk factors, including smoking, HPV infection, and genetic susceptibility. To address these issues, future research should focus on leveraging longitudinal cohort studies to dissect age-period-cohort effects and validate biomarkers like HPV viral load and p16/Ki-67 dual staining for risk stratification. Furthermore, it is crucial to conduct cost-effectiveness analyses of targeted screening strategies across diverse SDI settings, ensuring equitable access to preventive measures. Expanding HPV vaccination coverage and enhancing screening programs, particularly in low- and middle-income regions, will be vital in mitigating the growing burden of cervical cancer. As we anticipate increased incidence and mortality rates globally by 2050, comprehensive and region-specific intervention strategies are essential to achieve sustainable cervical cancer control.

## Conclusion

Despite a general downward trend in the incidence, mortality, and disease burden of cervical cancer worldwide, the disease burden remains high, with marked regional disparities, particularly acute in low- and middle-income regions. This severely hinders the WHO’s goal of achieving the 90–70-90 targets worldwide by 2030 to eliminate cervical cancer. The aging population trends further exacerbate prevention efforts, especially in high-SDI and high-middle-SDI areas, where there is an urgent need for the implementation and promotion of tobacco control policies. In low- and middle-SDI regions, including the broader implementation and promotion of HPV vaccination are essential. Given the trends of global aging and population expansion in low- and middle-SDI regions, the prospect of an increasing cervical cancer burden is dire. This underscores the urgent need for international collaboration to establish region-specific, multilevel precision intervention systems aimed at achieving sustainable global cervical cancer control objectives.

## Data Availability

The original contributions presented in the study are included in the article/Supplementary material, further inquiries can be directed to the corresponding authors.

## Footnotes

1
https://gco.iarc.who.int/tomorrow/en/dataviz/isotype
