# Supplementary material for: Global trends and future projections of cervical cancer burden: an integrated analysis of GBD 2021, UN population and WHO HPV vaccination data
Source: Front Public Health. 2026 Jan 26;14:1702186. doi: 10.3389/fpubh.2026.1702186 (PMC12883747; doi:10.3389/fpubh.2026.1702186)
Supplement: Supplementary file 1 [file Data_Sheet_1.pdf]

Supplement Materials

Global Trends and Future Projections of Cervical Cancer Burden: An Integrated Analysis of GBD 2021, UN Population and WHO HPV Vaccination Data

Authors:

Dongxuan Shao 1†*, Ping Wu1†, Huici Jiang3†, Zhijie Wang1*

1 Department of Gynecology and Obstetrics, Shanghai Eighth People's Hospital, Shanghai 200235, China.

2 Department of Gynecology and Obstetrics, Shanghai Fourth People's Hospital, School of Medicine, Tongji University, Shanghai 200434, China.

† Dongxuan Shao, Ping Wu, and Huici Jiang contributed equally to this manuscript.

* Correspondence:

Dongxuan Shao

Department of Gynecology and Obstetrics, Shanghai Eighth People's Hospital, Caobao road #8, Shanghai 200235, China.

Email address: shaodongxuan18@163.com

Jiezhi Wang

Department of Gynecology and Obstetrics, Shanghai Eighth People's Hospital, Caobao road #8, Shanghai 200235, China.

Email address: wangzhj1968@sina.com

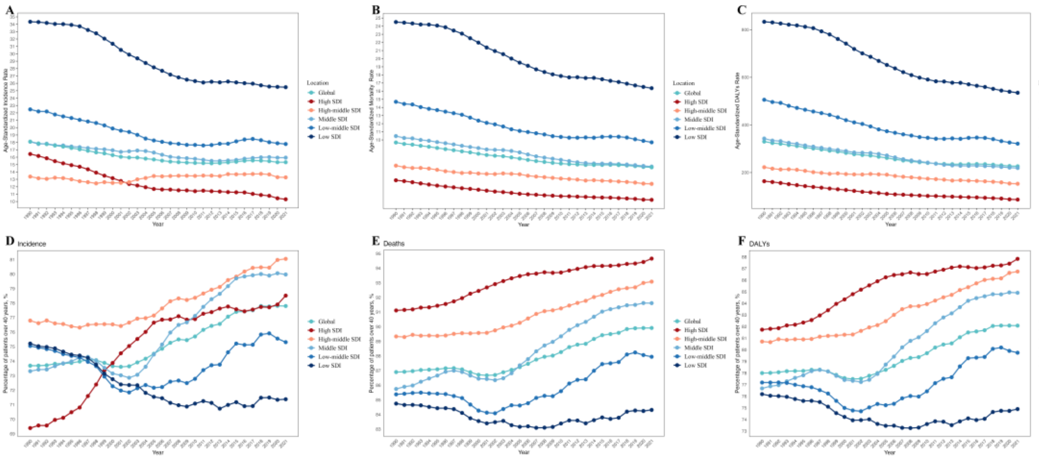

Fig.S1 Trends of ASIR (A), ASMR (B) and ASDR (C) in different SDI level regions from 1990 to 2021; trends of the percentage over 40 years of new cases (D), deaths (E) and DALYs (F) in different SDI level regions from 1990 to 2021.

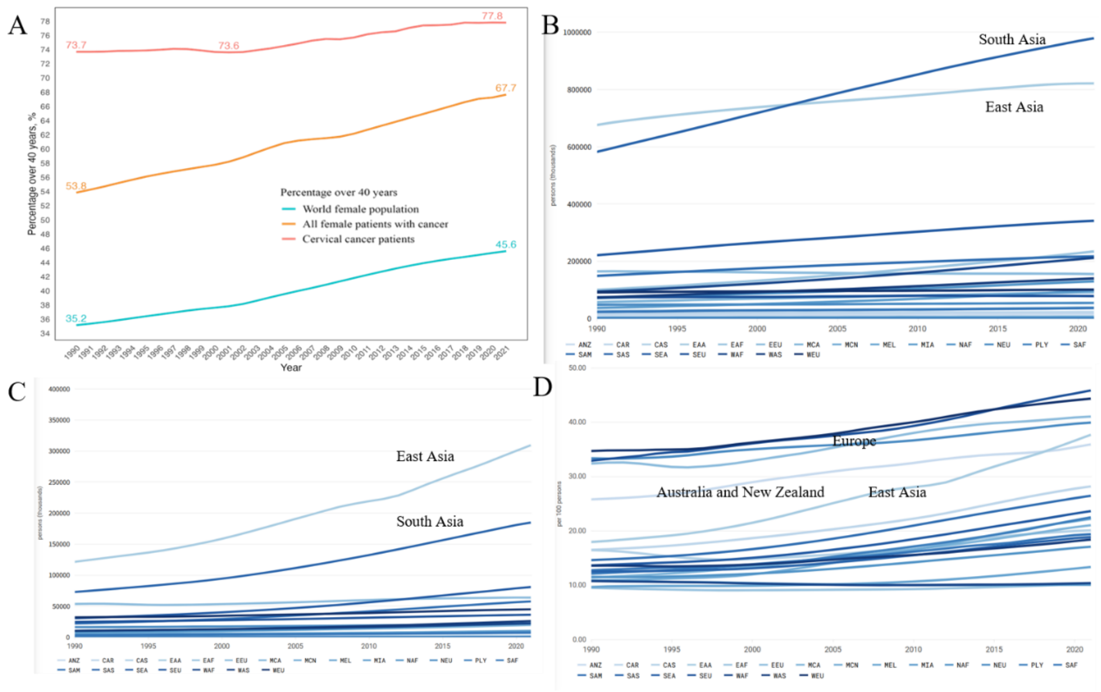

Fig.S2 Percentage change of population over 40 years by world female population (blue), all female patients with cancer (yellow), and cervical cancer patients (brown) from 1990 to 2021(A); Total female population change from 1990 to 2021 (B); Total female population change over 50 years (C); Percentage change of world female population over 50 years (D).

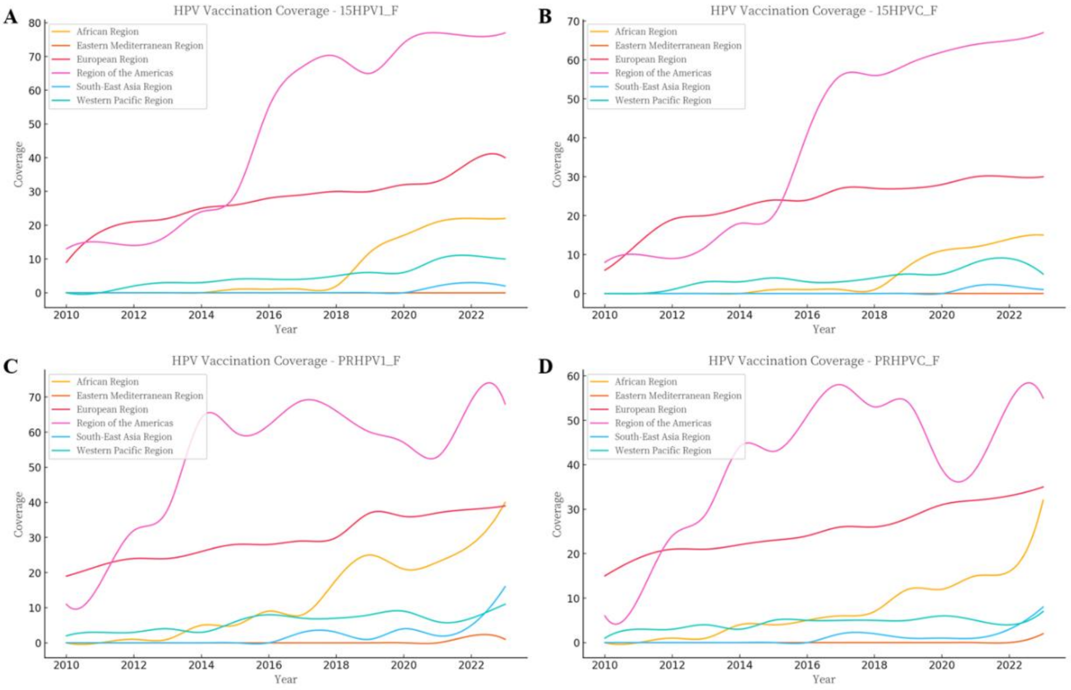

Fig.S3 HPV vaccination coverage by different schedule (15HPV first dose A, 15HPV last dose B, PRHPV first dose C, PRHPV last dose D) in different super regions.

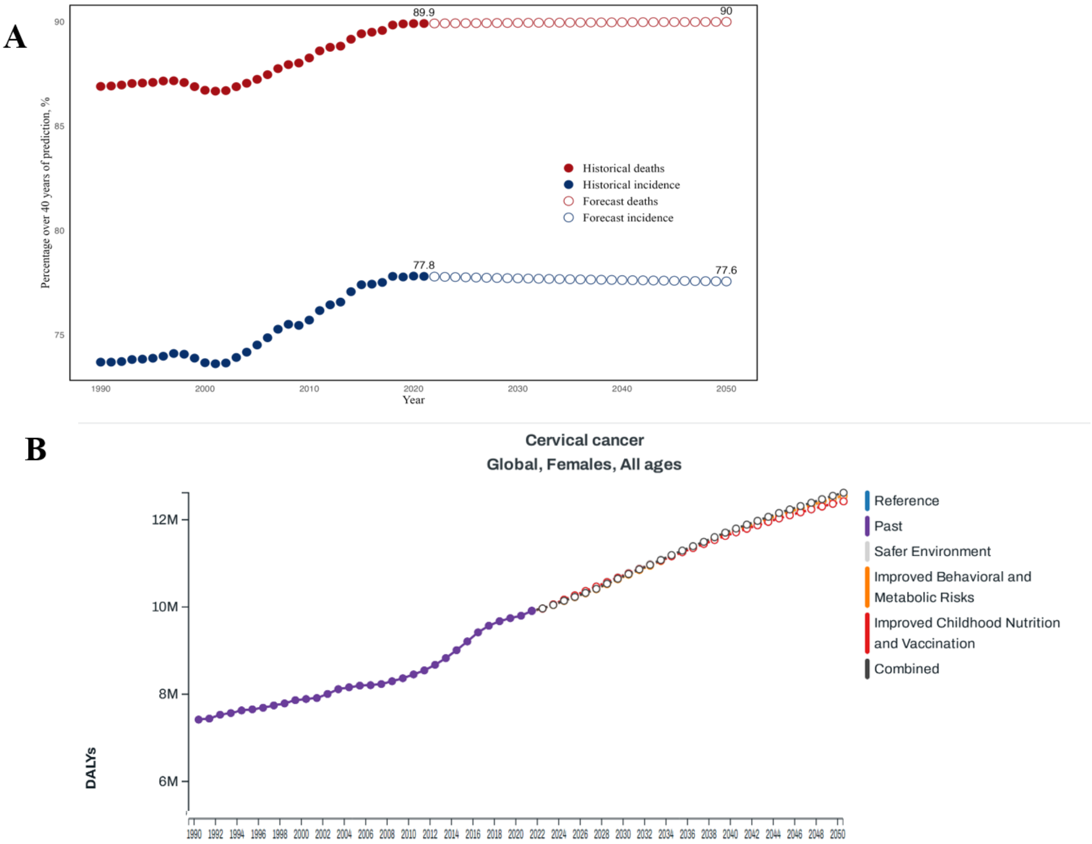

Fig. S4, The proportion of new cervical cancer cases and deaths in individuals aged

40 and above (A), as well as DALYs (B), with projections until 2050.

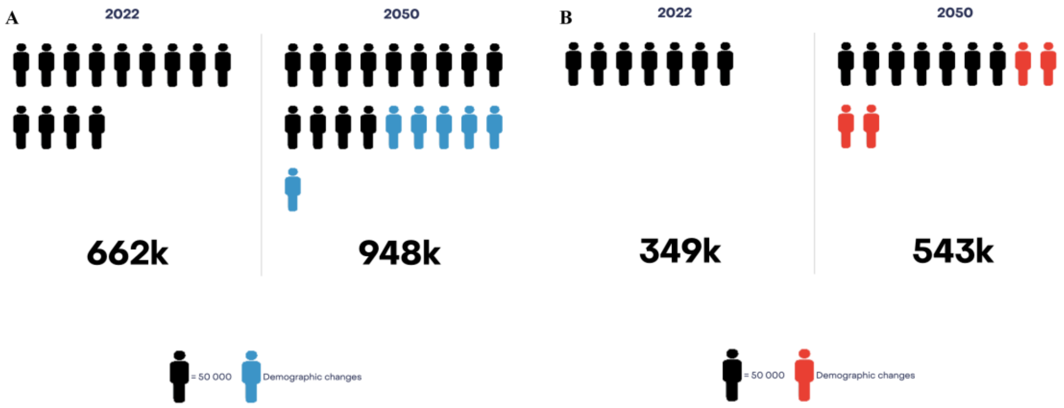

Fig. S5, The estimated number of new cases (A) and deaths (B) from 2022 to 2050 from International Agency for Research on Cancer

Table S1, New cases, deaths, and DALYs of cervical cancer in 1990 and 2021, by GBD regions and SDI level.

	New cases		Deaths		DALYs	
	1990	2021	1990	2021	1990	2021
Global	409,548	667,426	211,484	296,667	7,416,287	9,911,653
GBD regions						
East Asia	61909	137864	33633	52032	1178714	1616240
South Asia	83507	132482	52119	70315	1967172	2432061
Southeast Asia	30365	58017	16464	27513	613110	931501
Eastern Sub-Saharan Africa	22644	41370	14864	23270	564509	876849
Central Latin America	22434	40343	9518	13108	330665	441930
Western Sub-Saharan Africa	13972	34839	8886	19157	327427	709998
High-income North America	32695	30415	6834	7965	223497	240100
Western Europe	35616	27922	14370	10362	391715	257408
Tropical Latin America	13346	27823	6933	11759	242802	395392
Eastern Europe	23499	25339	12905	9929	378041	320236
Southern Sub-Saharan Africa	5528	16247	2941	8524	110032	300881
High-income Asia Pacific	12720	15578	4741	5074	144582	132808
Central Sub-Saharan Africa	6100	15328	4059	8913	150919	330691
Central Europe	16320	14203	8149	6590	263434	181664
North Africa and Middle East	6235	12913	3722	6268	137468	217737
Andean Latin America	4154	9757	2390	4435	83460	140301
Southern Latin America	6030	9302	3020	3832	103034	123079
Caribbean	4635	7382	2299	3397	82426	115954
Central Asia	5169	7179	2649	3093	89742	108285
Australasia	2001	1752	654	469	20693	13292
Oceania	668	1372	333	662	12844	25245
SDI level						
High SDI	73628	78008	29775	25893	888170	707650
High-middle SDI	84750	120153	37772	46301	1221304	1432108
Middle SDI	114367	228240	60363	96294	2165690	3167322
Low-middle SDI	48872	152720	51186	78178	1924362	2751152
Low SDI	73628	87633	32109	49687	1207230	1843208

Table S2, 92 countries or territories experienced an increase in incidence among the 15-39 age group from 1990 to 2021.

Countries or territories	SDI level	TPC of incidence	New cases in 2021
**Russian Federation**	**High-Middle**	**1.91**	**5328.25**

	**SDI**		
Lesotho	Low-Middle SDI	1.8	76.12
Kingdom of Eswatini	Low-Middle SDI	1.02	50.28
Zimbabwe	Low-Middle SDI	0.96	797.7
Italy	High-Middle SDI	0.93	472.88
Japan	High SDI	0.83	1762.66
**Bolivarian Republic of Venezuela**	**Low-Middle SDI**	**0.82**	**2281.11**
Namibia	Low-Middle SDI	0.81	66.33
**Brazil**	**Middle SDI**	**0.7**	**7877.12**
**China**	**High-Middle SDI**	**0.68**	**20970.03**
Trinidad and Tobago	High-Middle SDI	0.65	65.19
Thailand	Middle SDI	0.59	1894.62
Republic of Niue	Middle SDI	0.57	0.05
Sierra Leone	Low SDI	0.55	274.02
**Colombia**	**Middle SDI**	**0.54**	**2310.39**
**Pakistan**	**Low-Middle SDI**	**0.54**	**2625.96**
El Salvador	Low-Middle SDI	0.53	351.11
Samoa	Low-Middle SDI	0.52	6.73
Solomon Islands	Low SDI	0.52	20.93
Democratic People's Republic of Korea	Low-Middle SDI	0.51	498.8
American Samoa	High-Middle SDI	0.5	0.95
Liberia	Low SDI	0.48	184.86
Libya	High-Middle SDI	0.47	120.9
Congo	Low-Middle SDI	0.42	261.16
Jamaica	Middle SDI	0.41	138.84
Dominica	High-Middle SDI	0.41	2.46
Tokelau	Middle SDI	0.4	0.06
Marshall Islands	Low-Middle	0.39	2.31

	SDI		
Egypt	Low-Middle SDI	0.39	309.56
**South Africa**	**Middle SDI**	0.39	**3295.98**
Mongolia	Low-Middle SDI	0.38	69.27
Dominican Republic	Middle SDI	0.37	350.89
Djibouti	Low-Middle SDI	0.36	35.95
Republic of the Gambia	Low SDI	0.35	77.02
Belize	Low-Middle SDI	0.35	21.35
Bosnia and Herzegovina	High-Middle SDI	0.32	50.21
South Sudan	Low SDI	0.31	320.12
Republic of Côte d'Ivoire	Low SDI	0.29	353.57
Botswana	Middle SDI	0.28	65.28
Canada	High SDI	0.25	1022.25
Peru	Middle SDI	0.24	1184.39
Uganda	Low SDI	0.23	1322.21
Belarus	High-Middle SDI	0.23	195.07
Kenya	Low-Middle SDI	0.22	790.3
Saudi Arabia	High SDI	0.22	227.56
Suriname	Middle SDI	0.22	26.64
Equatorial Guinea	Middle SDI	0.22	54.04
Guyana	Middle SDI	0.21	30.75
Ecuador	Middle SDI	0.21	484.22
Guam	High-Middle SDI	0.2	2.06
Sao Tome and Principe	Low-Middle SDI	0.2	8.64
Uruguay	High-Middle SDI	0.19	134.86
**Argentina**	**High-Middle SDI**	**0.19**	**2082.44**
Paraguay	Middle SDI	0.18	344.46
Chad	Low SDI	0.17	399.59
Gabon	Middle SDI	0.17	57.21
Malawi	Low SDI	0.15	981.72
Turkmenistan	Middle SDI	0.14	98.89

Republic of Palau	High-Middle SDI	0.13	0.85
Togo	Low SDI	0.12	281.13
Malaysia	High-Middle SDI	0.12	498.87
Morocco	Low-Middle SDI	0.12	333.56
Kazakhstan	High-Middle SDI	0.12	470.48
Albania	High-Middle SDI	0.12	28.66
Vanuatu	Low-Middle SDI	0.1	7.32
Mauritania	Low-Middle SDI	0.1	97.18
Guatemala	Low-Middle SDI	0.09	556.12
Commonwealth of the Bahamas	High-Middle SDI	0.08	17.57
Principality of Monaco	High SDI	0.08	0.58
Tunisia	Middle SDI	0.07	96.7
Saint Vincent and the Grenadines	Middle SDI	0.07	6.15
Republic of Nauru	Middle SDI	0.06	0.55
Seychelles	High-Middle SDI	0.06	3.66
Guinea-Bissau	Low SDI	0.06	96.6
Kiribati	Low-Middle SDI	0.05	8.41
**Democratic Republic of the Congo**	**Low SDI**	**0.05**	**2521.08**
Chile	High-Middle SDI	0.04	664.84
Philippines	Middle SDI	0.04	1658.76
Senegal	Low SDI	0.04	415.57
Bahrain	High-Middle SDI	0.04	5.5
Puerto Rico	High-Middle SDI	0.03	46.99
Benin	Low SDI	0.03	290.63
Tonga	Middle SDI	0.03	3.19
Guinea	Low SDI	0.03	611.81

Eritrea	Low SDI	0.03	237.66
Central African Republic	Low SDI	0.03	184.21
Cameroon	Low-Middle SDI	0.02	1002.8
Angola	Low-Middle SDI	0.02	999.55
Costa Rica	Middle SDI	0.02	196.04
Bhutan	Low-Middle SDI	0.02	12.57
Federated States of Micronesia	Low-Middle SDI	0.02	3.18
Bulgaria	High-Middle SDI	0.01	176.41

(In summary, Countries counts, High SDI=4, High-middle =22, Low SDI=17, Low-middle=26, Middle=23. New cases count, High SDI=3013.05, High-Middle SDI=31339.13, Low SDI=8572.73, Low-Middle SDI=11273.37, Middle SDI=20175.22)
